# 

**Published:** 1891-12

**Authors:** 


					DfiCFijUBEK, 180 1.
THE'
-r+fc O F*i??
DENTAL SCIENCE.
EDITED BY
F. J. S. GOKGAS, M. p., D. D. S.
Vol. 35.
THIRD SERIES.
mo. s.
BALTI MORE
SNOW-DEN & COWMAN, 9 W. Fayette Street.
LON DO N :
TPIUBNE.R & CO., 60 Paternoster Row.
Entered at the Post Office at Baltimore, Md., as Second-class Matter.
PRYRMjE IN 21DV^NCE.
PUBLISHED MONTHLY
IS2.50 PER ilNNUM.

				

